# Patient-centred communication intervention study to evaluate nurse-patient interactions in complex continuing care

**DOI:** 10.1186/1471-2318-12-61

**Published:** 2012-10-11

**Authors:** Katherine S McGilton, Riva Sorin-Peters, Souraya Sidani, Veronique Boscart, Mary Fox, Elizabeth Rochon

**Affiliations:** 1 Department of Research, Toronto Rehabilitation Institute-UHN, E.W, Bickle Centre for Complex Continuing Care, 130 Dunn Avenue, Toronto, Ontario, M6K 2R7, Canada; 2 L. Bloomberg Faculty of Nursing, University of Toronto, 155 College Street, Toronto, Ontario, M5T 1P8, Canada; 3 Private Practice, Speech-Language Pathology, Thornhill, Ontario, L4J 5J2, Canada; 4 Daphne Cockwell School of Nursing, Ryerson University, 350 Victoria Street, Toronto, Ontario, M5B 2K3, Canada; 5 School of Health & Life Sciences and Community Services, Conestoga College Institute of Technology and Advanced Learning, 299 Doon Valley Drive, Kitchener, Ontario, N2G 4M4, Canada; 6 School of Nursing, York University, 4700 Keele Street, Toronto, Ontario, M3J 1P3, Canada; 7 Department of Speech-Language Pathology, University of Toronto, 160-500 University Avenue, Toronto, Ontario, M5G 1V7, Canada

**Keywords:** Aphasia, Communication intervention, Complex continuing care, Individualized communication strategies, Knowledge translation strategy, Nurse-patient interactions, Stroke

## Abstract

**Background:**

Communication impairment is a frequent consequence of stroke. Patients who cannot articulate their needs respond with frustration and agitation, resulting in poor optimization of post-stroke functions. A key component of patient-centred care is the ability of staff to communicate in a way that allows them to understand the patient’s needs. We developed a patient-centred communication intervention targeting registered and unregulated nursing staff caring for complex continuing care patients with communication impairments post stroke. Research objectives include 1) examining the effects of the intervention on patients’ quality of life, depression, satisfaction with care, and agitation; and (2) examining the extent to which the intervention improves staff’s attitudes and knowledge in caring for patients with communication impairments. The intervention builds on a previous pilot study.

**Methods/design:**

A quasi-experimental repeated measures non-equivalent control group design in a complex continuing care facility is being used. Patients with a communication impairment post-stroke admitted to the facility are eligible to participate. All staff nurses are eligible. Baseline data are collected from staff and patients. Follow-up will occur at 1 and 3 months post-intervention. Subject recruitment and data collection from 60 patients and 30 staff will take approximately 36 months. The Patient-Centred Communication Intervention consists of three components: (1) development of an individualized patient communication care plan; (2) a one-day workshop focused on communication and behavioural management strategies for nursing staff; and (3) a staff support system. The intervention takes comprehensive patient assessments into account to inform the development of communication and behavioural strategies specifically tailored to each patient.

**Discussion:**

The Patient-Centred Communication Intervention will provide staff with strategies to facilitate interactions with patients and to minimize agitation associated with considerable stress. The improvement of these interactions will lead to a reduction of agitation, which has the additional significance of increasing patients’ well-being, quality of life, and satisfaction with care.

**Trial registration:**

ClinicalTrials.gov Identifier NCT01654029

## Background

Whereas stroke is a leading cause of death in the United States and Canada, survival estimates are 75% - 85% in both countries [1,2]. About 10% of patients with disability post-stroke live in institutions [3]. More than 300,000 stroke survivors in Canada typically live with substantial and lasting physical and neuro-cognitive deficits [4,5], with 25-40% of them presenting with aphasia, the inability to produce or understand language [6]. These patients often are admitted to a long term care (LTC) facility because of their neurological disorders [7]. Up to 50% of LTC patients have speech and communication impairments [8]. When patients can neither articulate their needs nor be understood following a stroke, depression, agitation and behavioural symptoms often ensue [9-12].

Traditionally, nursing staff have treated behavioural symptoms exhibited by patients with stroke with restrictive interventions such as physical, environmental or chemical restraints [13], which are regarded as a major threat to patient care quality [14]. Additionally, caring for patients with behavioural symptoms contributes to staff burnout, which eventually leads to staff turnover, and negatively influences care quality [15]. Patient-centred care has emerged as a crucial underlying principle for the delivery of quality care in LTC facilities [16]. A key component of patient-centred care is the ability of staff to communicate in such a way that allows them to understand the patient’s needs. Research indicates that the occurrence of behavioural symptoms can be mitigated by the way nursing staff communicate with patients [17]. Therefore, enhancement of nursing staff’s communication strategies has been identified as a priority for LTC environments [18] and for maintaining patients’ quality of life [19]. However, two significant limitations of patient-centred care exist. First, the responsibility for ensuring the patient’s best care falls upon the nursing staff (both registered and unregulated care providers). However, many nursing staff lack the requisite specialized skills and abilities to effectively communicate with patients who have communication impairments and hence have difficulties understanding patients’ needs [8,20]. Providing assistance with personal care activities such as dressing, toileting and transferring requires frequent interactions between patients and nursing staff and if these interactions are compromised by communication breakdown and subsequent agitation, personal care interactions are disrupted and quality of care is undermined [19]. The end result is that the patient’s post-stroke functions and well-being are not optimized. Second, a recent systematic review of communication studies identified an absence of evidence-based interventions designed to enhance communication with patients with stroke living in LTC [21]. Improved staff communication with these patients is the basis for assessing patients’ needs and for providing patient-centered care.

Methodological and theoretical limitations across the studies that evaluated communication- focused interventions have left significant gaps in our understanding of how to effectively improve LTC staff’s communication skills. Approaches to communication training have been based on general recommended linguistic strategies [20,22]. Only one study had individualized tailored communication training [23]. Conversely, evidence is accumulating that communication enhancement strategies should be based on individualized patients’ remaining communication abilities [24,25]. Provision of effective and responsive care demands a focus on both communication and behavioural management strategies. How staff communicate with patients when a behavioural symptom occurs can influence patients’ behavioural symptoms [10,17].

To address some of these limitations, McGilton and colleagues [26] developed and pilot-tested a patient-centred communication intervention (PCCI) targeting nursing staff caring for patients with communication impairments post-stroke. The PCCI involved training by a Speech-Language Pathologist (SLP) to 18 nurses on 1 LTC unit on communication impairments and supportive conversation strategies. The SLP also developed individualized patient communication care plans based on initial assessments. The recent pilot work referred to as PCCI enhances quality of life, reduces agitation in patients and in turn creates more cooperative and less stressful caregiving situations for nurses [26]. The pilot study provided evidence to support the feasibility of a larger scale study to examine the efficacy of the PCCI, to determine its long-term effects, to test the intervention in new sites, and to determine its effects on a range of outcomes in a large sample of patients.

### Specific aims

This study implements the PCCI in Complex Continuing Care (CCC) with the objective to educate, train and support nursing staff in communicating effectively with patients who have communication impairments as a result of a stroke. The specific objectives include 1) to determine the effects of the PCCI on improved patient quality of life (communication and psychosocial domains), satisfaction with care, and agitation, at 1 and 3 months post-intervention; and 2) to examine the extent to which the PCCI improves nursing staff’s attitudes and knowledge about caring for patients with communication impairments at 1 and 3 months post-intervention.

## Methods/design

A quasi-experimental repeated measures non-equivalent control group design guides this study. In this design, control subjects from two units in one facility were enrolled and followed for up to three months, dependent on when they were discharged. Once the control subjects were accrued, staff from the facility were trained in the PCCI model. Currently the intervention cohort is being enrolled and followed.

Outcome data pertaining to the variables of interest are obtained from the patient participants in the control and experimental units at 3 different time points: 1) baseline, prior to delivering the intervention; 2) post-test 1, within 4 weeks after completion of the intervention; and 3) post-test 2, 3-months after completion of the intervention. Outcome data pertaining to the nursing staff are obtained at baseline and again at post-test 2 or prior to the patient’s discharge. Ethics approval was received from the Toronto Rehabilitation Institute Research Ethics Board #09-009 and from the research facility ethics board #2010-002-1002.

### Setting and sample

In order to accrue the required sample size, one of the largest CCC facilities in the Greater Toronto Area, Ontario, Canada, is participating in this study. In Ontario, CCC provides medically complex and specialized services over extended periods of time. Clients are usually admitted to a CCC unit following an acute care episode and are generally younger and have higher acuity needs than clients found in long-term care settings. Most clients will remain on these CCC units for an extended length of stay. CCC is often used interchangeably with “extended care” or “chronic care” [27]. The selected facility has approximately 300 patients and has 2 units with patients who have suffered a stroke. One unit has residents who stay for up to 1 month and the other unit has a length of stay closer to 3 months as patients have different levels of acuity, which will be accounted for in the analysis.

The sample consists of nursing staff (health care aides/personal support workers, registered practical nurses, registered nurses) and patients from the selected units. Eligibility criteria for staff include 1) are directly involved in providing care; 2) work full or part-time; and 3) consent to participate in the study. All staff members employed in the selected institutions are required to speak, read and write in English, and have the basic skills and knowledge to care for stroke patients.

Patients are invited to participate if they have been identified by the staff as having difficulties communicating. Specific inclusion criteria are 1) confirmed diagnosis of stroke, related to a cerebral infarct, as reported in the patient’s medical chart; and 2) English as the mother tongue or ability to sufficiently speak and understand English before the stroke as assessed by the staff on the unit and/or as reported by the family. The exclusion criterion ensures that patients can reliably self-report; it includes severe receptive aphasia (i.e. global aphasia and severe Wernicke’s aphasia). The facility speech-language pathologists assess newly admitted patients for their potential appropriateness for the study. A potential subject’s nurse will obtain permission from the patient and/or family to allow the research assistant to explain the study and consent process. If a participant is deemed unable to consent, an authorized representative will provide informed consent with the assent of the patient. A copy of the signed informed consent will be given to the participant.

### Intervention

The intervention consists of three components: (1) development of individualized communication care plans; (2) nursing staff attendance at a workshop focused on communication and behavioural management strategies; and (3) implementation of a staff support system. The intervention will take 2 months to implement.

#### Development of individualized communication plans

The SLP (co-investigator) assesses the communication abilities of each patient at baseline. This information is collected in two ways. First, staff nurses working with the patient are asked to complete the Montreal Evaluation of Communication Questionnaire for Use in LTC (MECQ-LTC) [24]. Second, the facility-SLP, who is provided with a stipend, completes standardized communication, cognitive and perceptual assessments for each patient enrolled in the study. The two SLPs also consult to ensure that the facility SLP capitalizes on their recommended communication strategies outlined to date.

Based on these assessments, an individualized one-page communication care plan is developed for each patient by the SLP. The communication plan, based on work by Genereux et al. [24], has the following sections: a) how to communicate and be with the patient; b) how the patient communicates; c) what the patient’s behaviours mean and d) patient’s habits to know to avoid communication problems, including topics of interest for discussion. These plans are refined with the assistance of the nursing staff and the facility SLP responsible for individual patients’ care. An example of a communication care plan used in the pilot study is presented in Table 1. A bedside communication kit is provided for each patient with individualized aids to assist in communication, such as pictures of various aspects of care, feelings, and health care personnel, as well as a whiteboard and pen to encourage the use of written cues and responses. Both the patient and the nurse are taught how to use the kit, and the teaching is reinforced by the facility SLP.

**Table 1:** Communication Care Plan

**How to communicate with patient**	**How patient communicates**
To help her understand you:	-has aphasia and some visual field impairment; ↓ attention to right side
- give her time to process what you’ve said	
	-understands short and more complicated instructions but needs ++ time to respond
To help her express herself:	
	- has limited speech; has hesitations and incomplete sentences; benefits from time to get her message out
-verify her yes/no responses (*use the yes/no sheet in the kit; have her point to yes/no)*	
	- her yes/no answers are not always accurate
	-wears glasses for reading
- ask her to point or gesture if she can’t say what she wants to express	-can read/understand written words and simple sentences; often needs to re-read to understand better
-or- give her written choices to point to *(use the whiteboard in the kit)*	-can write letters, numbers and simple words
-or-ask her to write a word or the first few letters of a word if she can’t say it *(use the whiteboard in the kit)*	
-make sure she’s wearing her reading glasses if using written cues	
-give her time to express herself	
Keep materials and written cues on LEFT side.	
**What patient behaviours mean**	**Patient habits to know to avoid communication problems**
-the words & phrases she repeats over and over may not be what she wants to say; use the strategies above to help her express herself.	- her communication is worse when she’s tired; discuss important issues when she’s rested.
	-in general, she needs extra time to understand you and to express herself
	-communication problems interfere with her family and social life
	-can’t use the phone because she’s not understood

#### Workshop

All staff nurses who consent to participate in the study have attended a one day workshop taught by two of the investigators. The workshop covered content on communication and behavioural management and focused on refining the communication plans.

1. Communication management: Nursing staff are trained to use communication strategies that promote the patient’s ability to communicate, and acknowledge and reveal the patient’s competence. The strategies include an application of the ‘Supported Conversation for Adults with Aphasia’ training program [28] developed by staff at the Aphasia Institute. This approach is based on the idea that the undamaged cognitive competence of individuals with communication disorders can be revealed through a trained conversation partner’s skill. Conversation partners, in this case nursing staff, are trained to acknowledge the patient’s competence using strategies such as natural adult talk and showing sensitivity to the person and context. They are also shown how to reveal patients’ competence by using strategies to ensure the patient understands what has been said and has a means of responding, and by verifying that this process has occurred. Nonverbal techniques that are used to acknowledge and reveal competence include gestures, writing, drawing, and using pictures and other resources. An adult learning approach was used to train nurses how to apply these strategies in practice [28-30].

2. Behaviour management: This component involves the use of the REAP (Relate well, Environmental manipulation, Abilities focused care and Personhood) model of practice [31]. The REAP model of care was conceptualized by the PI and has been used in other research with older adults. Staff nurses are taught that all behaviours have meaning, often related not to pathology but to unmet needs (e.g. physical, psychological/emotional, social, environmental) [32]. In other words, patients are exhibiting ‘responsive behaviours;’ that is, they are responding to such frustrations as discomfort, boredom, or loud sounds. Staff are encouraged to use the REAP model to determine the patient’s unmet needs and respond according to their assessment of these needs [31-37]. These strategies are also listed in the communication care plan. Four principles underlie the model:

i. Staff’s ability to relate well is an essential component of nursing staff-patient interactions [33,34]. Staff nurses are taught techniques to compensate for their patients’ impairments by using strategies found to be effective when patients are agitated such as calm voice, gentle touch, and calm approach.

ii. Environment-person theory[35] argues for the need for synergy between person and environment. The environment must be modified and changed to accommodate the person’s changing needs and preferences. For example, environmental noise should be reduced when communicating with a patient who is hearing impaired [36,37].

iii. Abilities focused care[37] involves staff focusing on patients’ retained abilities. The ability of persons with stroke to communicate is influenced by their spatial orientation, their hearing and vision, and their level of communication impairment. Staff nurses are informed by the SLP of the patient’s communication abilities and are taught to compensate where necessary.

iv. Personhood[38] refers to knowing the person, becoming familiar with the individual, and gaining knowledge of the person’s life. When providing care to patients, knowing what interests them helps to limit the patient’s behavioural symptoms [39]. Topics of interest (i.e., hobbies, families, etc.) for conversations that will engage the patient in meaningful interactions are obtained from the patient and family by the facility SLP and nursing staff.

#### Staff support system

The staff support system is an integral component of the PCCI. Following the workshop, the PI and the SLP trained the facility SLP on how to support staff to use the communication care plan in everyday practice. Supporting the staff at the bedside is a credible model that has been widely used in education research [40-42], and it is highlighted in the knowledge translation literature as useful in enhancing skill development and change [43,44]. In this study, supporting nursing staff involves providing positive feedback to nursing staff who acquire new skills, observing nursing staff interact with their patients, and providing teaching at the bedside when necessary. The facility SLP is asked to support each nursing participant for 1 hour a week for a three-week period after the workshop and to provide ongoing support. The SLP is available to consult with the facility SLP or educator as required.

### Data collection

The research assistant (RA) was trained in data collection procedures by the PI and SLP on general aging topics, stroke, communication impairments, and confidentiality/privacy issues. The SLP taught and demonstrated the procedures for obtaining self-report data and consent from patients with communication impairments. In addition, the training involved inter-reliability checks on the data collected. The RA is responsible for recruitment, data collection, and data management for the site.

#### Outcome measures

In order to maximize the likelihood of observing a significant effect of the PCCI, we selected outcome measures that demonstrated sensitivity to the intervention in our pilot work. The outcome measures for patient and the nursing staff are described.

#### Patient measures

##### Patient characteristics

We record age, sex, length of stay, education, date of stroke, and co-morbidities (the number of medical diagnoses other than stroke) from the patients’ medical records. The severity of patients’ communication disability as assessed by the Western Aphasia Battery (WAB) is considered as a co-variate; this patient characteristic has been found to influence staff-patient interactions [45].

##### Patient assessments of language and cognition

Language impairments (WAB), visuo-spatial impairment (Birmingham Object Recognition Battery, BORB), visual neglect (Bell’s test), and cognitive impairment (Mini-Mental State Examination, MMSE) are assessed. These data are used to determine the patient’s abilities and to determine the strategies the SLP recommends in the communication care plan.

1. The Western Aphasia Battery (WAB) is a comprehensive, multifactorial battery designed to evaluate a broad range of language impairments that often arise as a consequence of acquired brain dysfunction such as stroke.

2. Birmingham Object Recognition Battery (BORB) – Minimal Feature View Task provides a set of standardized procedures for assessing neuropsychological disorders of visual object recognition, based on tests developed from the cognitive neuropsychological literature. The Minimal Feature View Task assesses low-level aspects of visual perception (using same-different matching of basic perceptual features, such as orientation, length, position and object size and intermediate visual processes, e.g., matching objects different in viewpoint). The BORB has documented validity and reliability and provides normative data that are available for comparison [46].

3. The Bell’s Test is a cancellation test that permits a quantitative and qualitative evaluation of visual neglect which is important when staff attempt to communicate with the patient. It also provides valuable information on how the task is performed and the visual scanning pattern used by the participant. The Bell’s Test is sensitive for detection of mild and moderate neglect and permits a better exploration of the clinical manifestations of attention deficits in space [9].

4. The Mini-Mental State Examination (MMSE) is a widely used brief quantitative measure of cognitive status in adults. It has demonstrated validity and reliability in a variety of populations [47].

#### Patient outcome measures

The patient outcome measures below are collected by the RA. The *primary outcome* is the communication domain of the Stroke and Aphasia Quality of Life (SAQOL).

1. Patients’ quality of life is assessed with the SAQOL. The psychosocial domain, which includes 11-items (alpha of .67) and the communication domain with 7 items (alpha of .73) were selected as they are most responsive to the intervention. The communication domain has demonstrated convergent validity with the American Speech and Hearing Association Functional Assessment of Communication Skills for Adults (r = .55). The psychosocial domain demonstrated convergent validity with the General Health Questionnaire (r = .65) [48].

2. Patients’ satisfaction with care is measured with the Relational Care Scale (RCS)[49], which has acceptable internal consistency (Cronbach’s alpha = 0.87) and test-retest reliability (r = 0.70). The 6-item scale demonstrated sensitivity to change in two studies of patients with communication impairments [26,45].

3. Depressive symptoms are assessed with the 5-item Geriatric Depression Scale[50]. This is a well established measure that has been validated and used to assess depression in adults with aphasia following a stroke [51].

4. Agitation is assessed during nursing staff-patient interactions using the observational measure Pittsburgh Agitation Scale (PAS). The PAS rates the severity of agitation on a scale ranging from 0 to 4 in four general behaviour groups: aberrant vocalization, motor agitation, aggressiveness, and resisting care [52]. Reliability and validity testing of the PAS have been conducted in both acute geropsychiatric units (intraclass correlation coefficient [ICC] of .82) and nursing homes (ICC=.93) [52].

#### Nursing staff measures

##### Nursing staff characteristics

The following data are obtained from the nursing staff by the RA following a nurse-patient observation. Characteristics include age, sex, education level, job status, job category, and length of time employed on the unit. The job category of the staff will be used as a co-variate when analyzing staff outcomes, as staff’s knowledge of communicating with patients with communication impairments differs depending on job category [45]. Most staff members are willing to participate because of the endorsement of our research by administrators at the institution and based on previous recruitment rates for evaluation studies [26,45]. A sample size of 19 staff was sufficient to demonstrate changes in staff’s attitudes in the pilot study.

##### Nursing staff’s evaluation of the nurse-patient communication patterns

For the purpose of developing the individualized communication plans, each staff member is asked to complete the (MECQ-LTC) for the assigned patient. The MECQ-LTC is designed to (i) evaluate the frequency of use of various means of communication employed by the patient and his/her nurse; (ii) evaluate the number of different communication acts realized by the patient; (iii) determine the degree of effort required by nursing staff for efficient communication with a patient in diverse communication situations; and (iv) identify problematic communication situations [24]. The scale has established interrater-reliability and construct validity [24].

#### Nursing staff outcomes

1. Attitudes of the nursing staff toward communicating with patients with communication impairment are measured with the Communication-Impairment Questionnaire (CIQ), an 8-item self-report scale [24]. The measure focuses on the extent to which a staff member espouses a person-centred approach in relating to individuals with communication impairments. Reliability data from the pilot study indicated a Cronbach’s alpha coefficient of .73. In addition, the scale was sensitive to change following communication interventions [24,26].

2. Knowledge of staff working with patients with communication disabilities is measured using the Providers Interactional Comfort Survey (PICS)[20]. The scale has an acceptable reliability with a Cronbach’s alpha coefficient of .81 and has shown sensitivity to a communication intervention [45]. The measure focuses on nurses’ perception of their competence, confidence, willingness, frequency, and scope of practice related to interacting with patients.

#### Assessment of treatment fidelity

To assess for treatment fidelity it is necessary to ensure that procedures are in place to monitor for protocol adherence [53]. We monitor treatment fidelity by assessing staff’s adherence to the strategies outlined in the patient’s communication care plan. The strategies used during nurse-patient interactions are assessed by the Interaction Rating Form (IRF)[54], refined during our pilot work. The IRF includes operationalized observational ratings of strategies that are commonly suggested by SLPs when treating patients with communication impairments [26,54]. For example, items include: did the nurse use open ended questions or pictures, stand on the right side of the patient when communicating, and use gestures during the interactions. High scores indicate high levels of adherence to the treatment. Inter-rater reliability (r=.91-.95) and construct validity (r =.84 to .96 with clinical judgment) have been reported for the IRF [54].

### Statistical analysis

#### Sample size

The primary outcome for the patients is an improvement in the communication domain of the SAQOL (SAQOL-com) from pre- to post-test at 1 month. In our pilot study, the mean SAQOL-com score increased 3.8 units to 21.0 from a baseline score of 17.2 [26]. The baseline standard deviation was 5.0. This provides a Cohen’s standardized effect size of 0.76. A sample size of 44 is estimated to have 90% power to detect this effect using a repeated measures hierarchical regression analysis with an intra-class correlation of 0.40 among the three repeated measures and for an alpha of 0.05 [55].

The primary outcome for the staff is an improvement in the score from pre- to post-test in attitudes related to communicating with patients who have communication impairments (CIQ). In the pilot study, the improvement was 2.4 with a standard deviation of 4.1 providing a Cohen’s standardized effect size of 0.58. A sample size of 55 is estimated to have 80% power to detect this effect using a repeated measures hierarchical regression analysis with an intra-class correlation of 0.40 among the three repeated measures for an alpha of 0.05 [55]. We plan to recruit 60 patients: 30 in the control group and 30 in the intervention group. Each patient assessment will include a nurse-patient interaction; therefore, the subjects will be recruited as nurse-patient dyads.

#### Objective #1

The primary objective is to determine if the PCCI results in improved patient quality of life (communication and psychosocial domains) and satisfaction with care. The outcomes at each time point will be analyzed as repeated measures data using hierarchical regression methods to account for the nesting within the data. The patient outcomes at each time point will be measured for a specific patient by a specific staff member. The two levels of the model are expected to include (Level 1) repeated measures nested within (Level 2) patients. An appropriate model is expected to include Level 2 predictors, such as severity of patient impairment, as covariates and the effect of PCCI as the fixed effect of most interest. Because the PCCI will be specifically tailored to account for characteristics of the communication challenges of each individual patient, Level 2 predictors are expected to be an important part of the model. Characteristics of staff will be incorporated into the model by treating staff as a crossed random effect. The steps of building the model will be to 1) estimate the parametric structure of the covariance matrices; 2) model the structure of the mean responses by specifying the fixed effects; 3) specify the covariance structure within and among patients and staff; and 4) fit the mean model, accounting for the covariance structure. This will be repeated until an appropriate and parsimonious model is built. The results of most interest are the set of planned post-hoc contrasts that will identify the point in time when changes in the outcome occurred and whether the change in the outcome was maintained over time, increased with additional exposure to the improved patient-staff communication, or deteriorated over time. The expectations are that changes in the outcomes will occur at the first post-intervention time point and will be maintained throughout all subsequent time points. The analysis will be performed separately for each outcome measure.

#### Objective #2

The second objective evaluates the effects of the PCCI on staff’s attitudes and knowledge about caring for patients with communication impairments due to stroke. The same approach to analysis will be used to assess the effects of the PCCI on staff experiences as was used to assess the effects of the PCCI on patient quality of life. Staff attitudes and knowledge of care are now the outcomes measured at each time point and will be analyzed as (Level 1) repeated measures nested within (Level 2) patients using the hierarchical regression methods described in the previous paragraph.

#### Treatment fidelity

Descriptive statistics will be used to examine the frequency of the adherence to the treatment fidelity at the post-test data collection periods. A total score will be computed to quantify the number of specific communication strategies that are actually implemented for each patient at each occasion of measurement. The relationship between the number of strategies applied and patient outcomes at post-test at time 1 will be examined. This as treated analysis will supplement the intention-to-treat analysis in determining the effectiveness of the intervention. In the hierarchical model, this total score of treatment fidelity can be included as a Level 2 predictor.

## Discussion

This study will lead to the improvement of services provided to care for stroke patients living in CCC by enhancing communication between nursing staff and patients with communication impairment in everyday practice. Patient-centred care involves staff communicating effectively to understand patients’ needs, thus enhancing their well being and maintenance of autonomy. The PCCI also has the potential to reduce nursing staff burnout and reduce turnover in facilities. By improving communication, patients will become less agitated and caregiver interactions less stressful. The findings will serve as the basis for establishing guidelines for best practices in institutional settings. Study findings will have relevance for managers and policy makers because they will inform nursing and speech language pathology services for patients with communication difficulties in institutional care, which are transferable to other health jurisdictions.

Implementation of the PCCI is timely. This study is implementing the PCCI in CCC with the objective to educate, train and support nursing staff in communicating effectively with patients who have communication impairments as a result of a stroke. The significance of this research is that the PCCI will provide nursing staff with viable strategies to communicate effectively with patients post-stroke. When interactions are effective and patients’ needs are met, patients will be less agitated, and their quality of life will be optimized. Because agitation can create an inordinate burden on staff, its reduction has the additional significance of reducing the stress of nursing staff. Our research aims to improve the quality of life and health of older persons by understanding how to provide more effective health services to institutionalized elders.

### Trial status

The trial is now accruing during the intervention phase of the study. Progress to date includes the collection of data on all 30 control subjects; 16 intervention participants are enrolled with an end date of March 2013. Twenty-five staff nurses have agreed to participate in the study and have completed questionnaires.

## Abbreviations

CCC: Complex Continuing Care; LTC: Long-Term Care; MECQ-LTC: Montreal Evaluation of Communication Questionnaire for Use in Long Term Care; PCCI: Patient-Centred Communication Intervention; SAQOL: Stroke and Aphasia Quality of Life; SLP: Speech Language Pathologist.

## Competing interests

The authors declare that they have no competing interests.

## Authors’ contributions

KM obtained funding for the study, is the Principal Investigator, and created the first draft of the manuscript. All co-investigators contributed to the study design. SS contributed the statistical analysis approach. All co-authors reviewed the manuscript, made substantial editorial changes, and approved the final manuscript.

## Pre-publication history

The pre-publication history for this paper can be accessed here:

http://www.biomedcentral.com/1471-2318/12/61/prepub
